# Sexual Dimorphism of Metabolite Profiles in Pigs Depends on the Genetic Background

**DOI:** 10.3390/metabo11050261

**Published:** 2021-04-22

**Authors:** Manuela Peukert, Sebastian Zimmermann, Björn Egert, Christoph H. Weinert, Thomas Schwarzmann, Dagmar A. Brüggemann

**Affiliations:** 1 Department of Safety and Quality of Meat, Max Rubner-Institut, 95326 Kulmbach, Germany; Sebastian.Zimmermann@mri.bund.de (S.Z.); Dagmar.Brueggemann@mri.bund.de (D.A.B.); 2 Department of Quality and Safety of Fruit and Vegetables, Max Rubner-Institut, 76131 Karlsruhe, Germany; Bjoern.Egert@mri.bund.de (B.E.); Christoph.Weinert@mri.bund.de (C.H.W.); 3 Staatsgut Schwarzenau, Leistungsprüfungsanstalt für Schweinezucht Schwarzenau (LPA), 97359 Schwarzenau, Germany; Thomas.Schwarzmann@baysg.bayern.de

**Keywords:** GC × GC qMS, blood, muscle, liver, pigs, meat quality

## Abstract

The study aimed to investigate possible systematic effects in the basic underlying variability of individual metabolomic data. In this context, the extent of gender- and genotype-dependent differences reflected in the metabolic composition of three tissues in fattening pigs was determined. The 40 pigs belonged to the genotypes PIx(LWxGL) and PIxGL with gilts and boars, respectively. Blood and tissue samples from *M. longissimus dorsi* and liver were directly taken at the slaughtering plant and directed to GC × GC qMS metabolite analysis. Differences were observed for various metabolite classes like amino acids, fatty acids, sugars, or organic acids. Gender-specific differences were much more pronounced than genotype-related differences, which could be due to the close genetic relation of the fattening pigs. However, the metabolic dimorphism between gilts and boars was found to be genotype-dependent, and vice versa metabolic differences between genotypes were found to be gender-dependent. Most interestingly, integration into metabolic pathways revealed different patterns for carbon (C) and nitrogen (N) usage in boars and gilts. We suppose a stronger N-recycling and increased energy metabolism in boars, whereas, in gilts, more N is presumably excreted and remaining carbon skeletons channeled into lipogenesis. Associations of metabolites to meat quality factors confirmed the applicability of metabolomics approaches for a better understanding about the impact of drivers (e.g., gender, age, breed) on physiological processes influencing meat quality. Due to the huge complexity of the drivers-traits-network, the derivation of independent biomarkers for meat quality prediction will hardly be possible.

## 1. Introduction

Quality traits such as tenderness, color and water-holding capacity (WHC) are of major importance for consumer acceptance, and therefore for the economic value of the meat. Even though the impact of animal handling and post-mortem processing is well described, the biochemical processes behind, which are affecting the meat quality traits, are still not fully understood. There is also an interest in the rapid prediction of meat quality in the meat production sector by using biomarkers [1,2,3]. Even though attempts have been made to predict meat quality by using various techniques like transcriptomics, proteomics or metabolomics, it appears that the complexity of meat quality can hardly be anticipated.

The principal processes affecting meat quality during early post-mortem metabolism are the rate and extent of glycogen degradation, anaerobic glycolysis and consequently the pH decline [4,5,6]. During the ripening phase the meat becomes tender as a consequence of proteolytic activity of several proteases, and activity of proteolytic enzymes depends on early post-mortem biochemical processes like pH development [7]. Therefore, protein biochemistry in relation to tenderization and juiciness was the main focus studying intramuscular post-mortem changes in the past [8,9,10,11]. In addition, research on muscle fiber biology and the interaction with intramuscular fat and connecting tissues has revealed insights into the complexity of the muscle to meat conversion [12,13,14]. Meanwhile, genomics approaches [15] and biochemical profiling approaches (proteomics, metabolomics) are additionally used to relate quality traits to particular biomarkers, and to look for the impact of metabolic networks [16,17,18]. So far, the broad analysis of metabolites has revealed several biomarkers for particular aspects of meat quality in chicken, beef and pork, but also for animal growth performances. In addition, the differentiation of species by meat metabolite profiling has been shown [19]. Regarding meat quality, metabolites from muscle catabolic pathways and oxidative stress response could be related to high ultimate pH in chicken [20]. Welzenbach et al., 2015 [21] found significant correlations of pyruvic acid, methylglyoxal and glucosylceramide to drip loss in pork. The high relation of drip loss and energy metabolism is obvious, but the relationship between drip loss and transformation products of sphingolipid metabolism hints towards cell stress and membrane degradation processes [21]. Bovo et al., 2016 [22] conducted a study on the genetically separated genotypes Italian Duroc and Italian Landrace using blood from exsanguination. The baseline metabolite profiles differed between both genotypes as obtained by multivariate statistical approaches. However, strong significant differences were only observed for a few metabolites. Carmelo et al., 2020 [23] applied an untargeted metabolomics approach to better understand the association of metabolite changes during maturation with nutrient utilization. Despite some linear predictability between timepoints being observed, they clearly emphasized the complexity of this trait because of remarkable differences between breeds and time points. In beef, a metabolomics approach was used to investigate the impact of the feeding type—grass vs. grain. Besides the nutritional differences of the meat (lipid profile, omega3/omega6 ratio), the authors found strong evidence for less stress in grass fed animals compared to grain fed animals [24]. In general, one objective of using OMICS technologies (transcripts, proteins or metabolites) in meat science is to precisely predict meat quality traits by deriving independent biomarkers [3,15,25,26,27,28]. However, the various laboratory approaches aiming at identifying generalized mechanisms underlying meat quality traits clearly showed that the impacts of gender, genotype and feeding are notable, which indicates that each modification/change in the biological system (pig) directly impacts metabolite levels resulting in inter-individual variations.

Techniques used for metabolite profiling approaches are nuclear magnetic resonance spectroscopy (NMR) and mass spectrometry (MS). MS offers a broad variability of analytical approaches depending on ionization technique and mass analyzer, for example quadrupole (q) or time of flight. Usually, a separation technique is used in front of a mass spectrometer, which can be gas chromatography (GC), liquid chromatography (LC) or capillary electrophoresis (CE). Each technique coming with its own pros and cons. The huge challenge in metabolite profiling, independent of the used technique, is to find compromises for the detection between low and highly concentrated compounds, between the various chemical properties of compound classes, and for a practicable separation in front of the MS. Meaning, no technique is able to detect everything, but only a small snap shot of the whole metabolome. In our study, we applied a comprehensive, two-dimensional GC × GC qMS approach, which provides a much better separation of analytes compared to 1D GC-MS, and therefore a much higher number of detected features could be used in statistical analysis [29,30]. Typical metabolites measured with GC-MS are amino acids, fatty acids, small organic acids, sugars and various derivatives, various heterocyclic compounds like the nitrogen bases (e.g., purines or pyrimidines) and sterols among others. 

In Germany, the most common fattening pigs are crossings of Piétrain (PI) as terminal sire with crossbred Large White (LW) × German Landrace (GL) or purebred GL on the mother side. The crossings are known for high lean meat percentage as well as a bigger eye muscle. Since the description of the mutation in the ryanodine receptor in 1991 [31] pig breeding has endeavored to produce a stress-stable pig population with a high proportion of lean meat. In Bavarian herdbook breeding the pig populations for landrace and Large White are 100% stress stable, the remediation of stress stability in the Piétrain population is not yet completed. However, less than 1% of the Piétrain population is homozygously mutated in the ryanodine receptor gene.

The objective of this study was to investigate the fundamental underlying variability in metabolomic data between individual animals, and to elucidate gender- and genotype dependent characteristics in metabolite profiles. Information about the extent of variation among different sample groups would be essential when relating metabolite profiles to meat quality attributes, and searching for independent biomarkers. We used sticking blood and hot muscle and liver tissues from two crossbred pigs, (PIx(LWxGL) and PIxGL), to depict the metabolite profiles before muscle had converted to meat.

## 2. Results

### 2.1. Meat Quality Data

**Figure 1:**
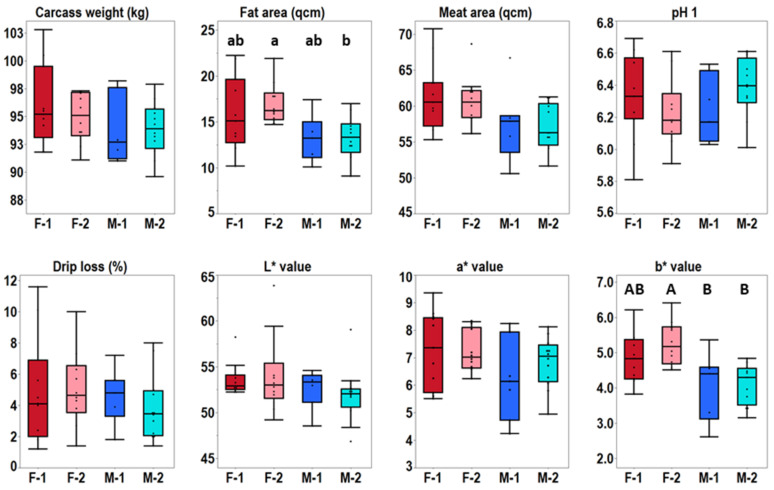
Meat quality data. Statistical differences were calculated using Tukey’s HSD Test. Significant differences are indicated with capital letters: *p* < 0.01 and lower-case letters: *p* < 0.05. Abbreviations: F-1 = female PIx(LWxGL), F-2 = female PIxGL, M-1 = male PIx(LWxGL), M-2 = male PIxGL.

The pigs were reared in the test station for about 100 days up to a carcass weight of approximately 95 kg. Carcass and meat quality data were recorded directly at the slaughtering plant and contained pH1, color and drip loss among other data (Figure 1, Supplementary Table S1). The meat quality parameters for three of the animals pointed towards impaired water holding (Supplementary Table S1). A negative correlation of pH1 and drip loss was determined (r = −0.69; *p* < 0.0001), which is in accordance with general meat physiochemical properties. Genotype-specific differences for the recorded meat quality data were not observed. Gender-specific differences were only observed for PIxGL crossbreds. The PIxGL gilts showed a significantly higher fat area (*p* = 0.0248) compared to boars. Interestingly, the PIxGL crossbreds also showed higher b* values (*p* = 0.0022) in gilts.

### 2.2. High Individual Variability of Metabolites in Muscle, Liver and Blood

A comprehensive GC × GC qMS approach was applied to extracts from muscle (*M. longissimus dorsi*), liver and total blood to analyse the basic individual metabolic variation of gilts and boars, and to elucidate if gender specific differences are influenced by the genotype. For all three tissues more than 300 features were used in statistical analyses (Table 1), respectively.

**Figure 2:**
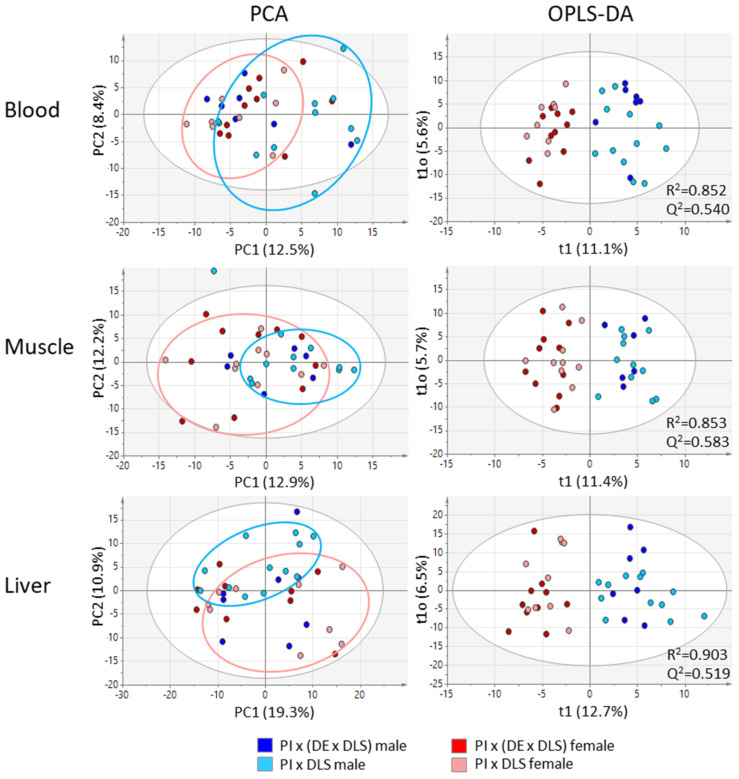
Principal Component Analysis (PCA) and Orthogonal Projections to Latent Structures Discriminant Analysis (OPLS-DA) score plots of metabolic profiles of blood, muscle and liver from two common German crossbreds and including female and male pigs. Data were mean centred and unit variance scaled; the white circle represents the 95% confidence interval.

The Principal Component Analysis (PCA) revealed high inter-individual variations between animals from one group (genotype-gender combination). In addition, a low variance among sample groups was observed by unbiased PCA (Figure 2). Neither the closely related genotypes nor the genders caused a clear differentiation in the PCA score plots. Data points for gilts (dark and light red) and boars (dark and light blue) largely overlapped indicating that the global metabolite profiles are based on individual characteristics. For PIxGL (light blue and light red), a small tendency of variation between gilts and boars could already be recognized and is indicated by circles.

According to the small variance between female and male pigs at a first sight, we applied in a second step supervised Orthogonal Projections to Latent Structures Discriminant Analysis (OPLS-DA). Application of OPLS-DA classification to all samples revealed clearer segregations between female and male pigs and acceptable qualities of the models were indicated by *R*^2^ values > 0.85 and *Q*^2^ values > 0.51 by using two components (Figure 2). No distinctive variation was observed in the vertical dimension that displays the within group variation (in our case between the two genotypes within gilts and boars), indicating again that differences between individuals strongly, affect the distribution of data points in the score plot. An OPLS-DA for the discrimination of both genotypes did not result in reliable models. We also checked for a sampling day-dependent variation but did not obtain an effect in blood. Differences observed for muscle and liver tissue showed no considerable effects for the parameters gender and genotype included in this study.

Potential differential features from OPLS-DA analysis were selected by Variable Importance Projection (VIP) (Supplementary Table S2) with features having a VIP > 1 considered to be influential. In addition, significance levels were pairwise calculated with Tukey’s HSD Test for normal distributed data and Steel-Dwass test for not normal distributed data (both tests correcting for family wise error rates). The selection of either parametric or non-parametric testing was based on the Anderson-Darling test for normality. In Table 1 an overview of the numbers of features with potential impacts on sexual metabolic dimorphism as well as genotype differentiation is presented. Even though the visualization of global metabolic profiles by PCA did not show a clear distinction of the genders and genotypes, the univariate statistical tests revealed a considerable number of diversifying molecular features. In accordance with the results from OPLS-DA, the number of significant gender specific features dominate compared to a few genotype specific features.

### 2.3. Accumulation of Gender Regulated Metabolites Is Genotype-Dependent

**Figure 3:**
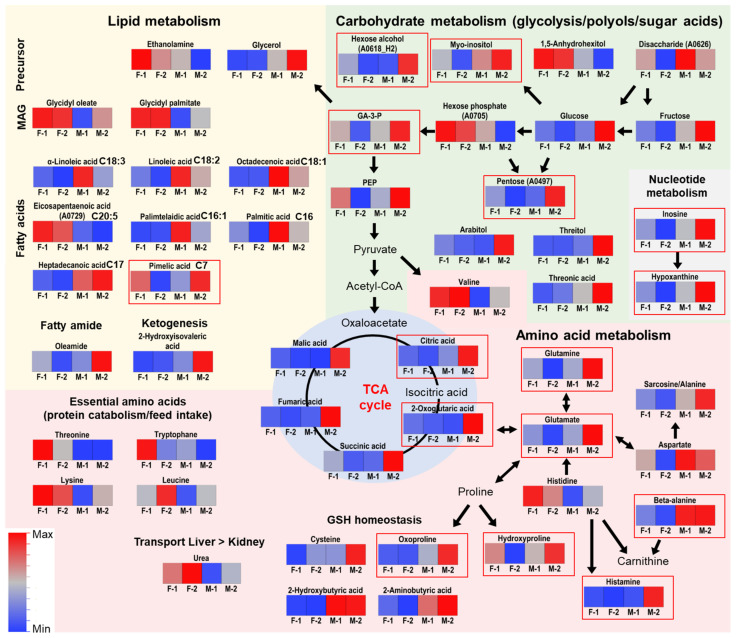
Pathway integration of varying metabolites in blood. Relevance of metabolites was determined by univariate statistical testing and OPLS-DA. Metabolites illustrated in red frames were found to be significantly different between boars and gilts (as observed by Tukey’s HSD Test or Steel-Dwass Test). Visualized are the unit variance scaled means of the respective signal intensities displayed as color coded heatmaps.

In order to identify molecular features whose abundances are gender-dependently regulated we compared gilts and boars from both genotypes. In this way, we determined features that showed a similar regulation in both genotypes, and those features that were genotype-specific regulated. For example, this was the case for N-acetylhexosamines in liver which were remarkably high in PIxGL gilts. A database search using the NIST library allowed for the annotation of putative biomarkers (Table 2), and integration into related biochemical pathways they are involved in (Figure 3). The differences affected various compound classes participating in a number of metabolic pathways including amino acids, lipids, organic acids or sugars. In addition to the significant features observed by Tukey’s HSD Test and Steel-Dwass Test, a number of compounds were observed by a simple Students T-Test and Wilcoxon Test, or were only observed by VIP scoring in OPLS-DA (Supplementary Table S2). These features were treated as tentative candidate compounds for male-female distinction.

#### 2.3.1. Divergences in the Free, Proteinogenic Amino Acid Pool

Amino acids that can only be degraded via the glucogenic pathway showed a mixed pattern. Glutamine, glutamic acid and aspartic acid were higher accumulated in boars, especially in the PIxGL line (Table 2). Glutamine, which connects amino acid metabolism between various organs was increased up to 66% and 46% in blood and muscle tissue from PIxGL boars compared to the gilts, respectively. In addition, glutamate was also increased by 35% in boar blood. The AAs valine and histidine were tentatively higher abundant in gilts (Supplementary Table S2).

**Figure 4:**
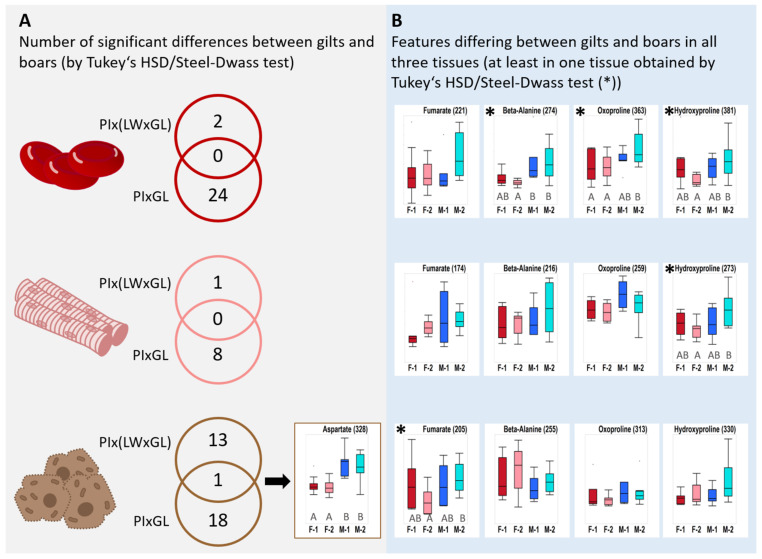
Gender-dependent differences in both genotypes. (**A**) Only significant analytes were summarized (obtained by Tukey’s HSD or Steel-Dwass testing); Aspartate is the only analyte significantly different in both genotypes. (**B**) Only those molecular features are illustrated in Box plots that were recorded in all three tissues as a candidate feature and showed at least in one tissue a significant difference (*). Significances (*) were only observed for the genotype PIxGL and are indicated with capital letters. No label means detected by VIP scoring and/or Student’s T-Test/Wilcoxon Test. The *y*-axis corresponds to total ion counts and is scaled to highlight the variation within and among groups. Abbreviations: F-1 = female PIx(LWxGL), F-2 = female PIxGL, M-1 = male PIx(LWxGL), M-2 = male PIxGL.

Liver aspartate levels in boars exceeded the levels in gilts by ca 65% in both genotypes. Aspartate was found to be the only compound significantly different between boars and gilts in both genotypes, which makes it a possible candidate as an independent biomarker for sexual metabolic dimorphism (Figure 4). In all three tissues, amino acids that are degraded via the ketogenic pathway (lysine, leucine) showed tentatively higher levels in gilts.

#### 2.3.2. Amino Acids with Crucial Physiological Importance Accumulated in PIxGL Boars

For all three tissues of PIxGL an influence of the gender was observed for the non-proteinogenic amino acids beta-alanine, 5-oxoproline and hydroxyproline (Figure 4). All three compounds are described to be involved in protective functions such as ROS scavenging and tissue protection and turnover. In particular, hydroxyproline showed significant higher levels in boar blood and muscle. The effects were least pronounced in liver and only recorded by VIP scoring (Supplementary Table S2).

#### 2.3.3. Increased Levels of Metabolites from Energy Metabolism in PIxGL Boar Blood

In addition, the citric acid cycle seems to be influenced by a metabolic sexual dimorphism in PIxGL (Figure 3). In all three tissues, fumarate was accumulated in boars (Figure 4). In blood samples, also citrate and 2-oxoglutarate differed significantly (Table 2), and malate and succinate were qualified by T-Test/Wilcoxon test and OPLS-DA (Supplementary Table S2). Citrate and malate were additionally detected as VIPs in muscle, and malate in liver. Gender differences in the TCA cycle as a central linker of metabolic pathways, and a central unit in energy metabolism, are stronger reflected in blood than in muscle or liver cells. In addition, glycerinaldehyde-3-phosphate (GA3P, to 76%) and phosphoenolpyruvate (to 68%) from the glycolysis pathway were accumulated more in PIxGL boars (Table 2 and Supplementary Table S2). Furthermore, in the same tissue, differences occurred in the adenosine nucleotide degradation pathway. Hypoxanthine was doubled in its signal intensity in PIxGL boars compared to gilts, and inosine was not at all detected in PIxGL gilts, but in boars (Table 2).

#### 2.3.4. Relationship between Markers of Increased Energy Metabolism and Amino Acids with Scavenging Functions

Pearson’s correlation coefficients for the above mentioned markers as well as their important precursors glutamine and glutamate showed that citrate was associated with levels of ß-alanine (r = 0.57), oxoproline (r = 0.67) and hydroxyproline (r = 0.50) (Table 3). The correlation coefficient was even higher between the two precursors and the first TCA cycle intermediate. In addition, the amounts of hypoxanthine seem to be related to the amounts of ß-alanine (r = 0.55). The high correlation of citrate and 2-oxoglutarate to hypoxanthine demonstrates the tight relation between the TCA cycle and ATP/ADP breakdown. In addition, we included components of GSH regulation in this analysis (Cysteine, 2-aminobutyric acid and 2-hydoxybutyric acid). All three components were strongly correlated with each other, and cysteine levels correlated also to hypoxanthine (r = 0.52).

#### 2.3.5. Enhanced Lipid Metabolism in Gilt’s Liver

Liver fatty acids showed a diverse pattern in both genotypes. In PIx(LWxGL) eicosatrienoic acid was barely detected in gilts but showed a much higher abundance in boars. In contrast, eicosapentaenoic acid was much lower in PIxGL boars than in gilts. Considering also the VIPs, a general trend to higher amounts of free fatty acids in gilts could be recognized (Supplementary Table S2). In addition, monoacylglycerols (MAG), which are intermediates in the breakdown and synthesis of triacylglycerols (TAG), showed a higher accumulation in gilts compared to boars. Fatty amides are a diverse group of metabolites depending on the fatty acid and the linked amino moiety. Two of these N-acyl amides detected in liver had highest levels in PIxGL gilts.

In muscle, no generalizable patterns were observed. For some compounds higher levels were observed in boars, like stearic acid or arachidonic acids (both saturated), whereas gilts showed higher levels for hexadecenoic acid (Supplementary Table S2). A similar mixed pattern could be seen in levels of MAGs and presumable precursors.

#### 2.3.6. Summary

It can be summarized that features contributing to sexual metabolic dimorphism are not generalizable but genotype-dependently pronounced (Figure 3). Interestingly, in PIxGL more differentiating features were found in all three tissues than in PIx(LWxGL) (Table 2), and only one genotype independent marker in liver could be observed. Furthermore, blood and muscle metabolites showed more similar trends, whereas in liver the differentiating compounds were more related to lipid metabolism. Nevertheless, it has to be considered that tissues for liver and muscle analyses were taken ca 30 min post-mortem so that during this time frame no oxygen and nutrient supply or metabolic exchange with other organs was possible, and resulting metabolite profiles were accordingly affected (might not represent in vivo conditions).

### 2.4. Differences According to the Genetic Background

**Figure 5:**
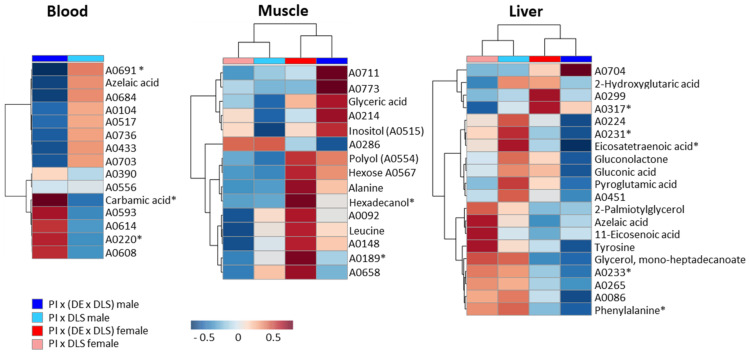
Heatmaps of features varying between genotypes. Displayed are the normalized (median normalization, unit variance scaled) ion intensities for relevant molecular features. Features that were significantly different by Tukey HSD/Steel-Dwass Test are labelled with [*]. All other features were obtained by Students T-Test/Wilcoxon Test, and are designated as most likely different. Supplementary Table S2 contains the corresponding values for log2 fold changes and *p*-values.

Among the multitude of measured analytes we found some that showed significant differences between the genotypes. Compared to the variation between males and females, the genetically-dependent variability was less pronounced (Table 1). Furthermore, these differences were confounded with gender effects, and were either detected in gilts or boars of the two crossbreds (Figure 5, Supplementary Table S2). In blood no distinguishing feature was observed between gilts of both genotypes, and 15 features were found to vary between boars (as observed by T-Test/Wilcoxon Test), three were significantly different (as observed by Tukey’s HSD Test/Steel Dwass Test). In muscle eight features showed a variation between gilts of both genotypes, of them two were significant, and seven features in boars varied to a small extent. Most varying features were detected in liver with six signals between gilts, one of them with a significant difference, and 14 signals between boars with four significantly different signals.

As has been revealed for gender-depending differences, the diversifying compounds between genotypes belonged to various metabolite classes. Additionally, these compounds were individual for each tissue. From the heatmap, it can be recognized that, in muscle tissue, more differentiating features (in gilts and boars) were found in the genotype PIx(LWxGL), whereas, in liver, this was the case for PIxGL. According to this low number of genotype-dependent variables, a conclusion about particular involved metabolic pathways could not be deduced.

### 2.5. Correlations of Candidate Compounds to Meat Quality Parameters

In order to check if meat quality parameters are associated to metabolite markers, and if they could be predicted with them we calculated Pearson correlation coefficients (Table 4) and multiple regression coefficients. Negative associations of the energy metabolites citric acid, inosine, GA3P and hypoxanthine to meat colour were observed for blood. In addition, muscle citric acid and liver fumaric acid were negatively correlated to meat colour. Furthermore, drip loss and pH 1 strongly correlated to meat citric acid and liver fumaric acid, which confirmed the huge importance of energy metabolism during early post-mortem processes. Interestingly, 1,5-Anhydroglucitol in muscle showed strong correlations to drip loss, L* and pH1, which supports the role of sugar metabolism on water holding. Similarly, liver acetyl glucosamine levels, which correlated to L*, drip loss, pH1 and fat area can be suspected to mirror the energetic status of the liver. The amino acid aspartic acid showed a strong association with b* in liver. The fat area correlated to several liver metabolites among them fatty acids, which seems obvious and implies the regulatory role of the liver for lipid metabolism.

For the muscle metabolites with correlations to meat quality parameters we also found corresponding associations to blood levels. 1,5-Anhydroglucitol levels in blood and muscle strongly correlated to each other (r = 0.94, *p* < 0.001). In addition, glutamine (r = 0.55, *p* < 0.001) and hydroxyproline (r = 0.54, *p* < 0.001) levels were associated as well as citric acid levels (r = 0.44, *p* = 0.008). 

For metabolite markers with at least a moderate Pearson correlation coefficient (r > 0.4) we calculated multiple regression coefficients to check for predictability of selected quality parameters (Table 4). Moderate predictability, *R*^2^ > 0.3 (according to Cohen 1988 [32]) was achieved for the parameters % drip loss, b* and fat area, involving different metabolite markers in all three tissues.

## 3. Discussion

### 3.1. Differences in Metabolite Levels between Boars and Gilts Are Genotype Dependent

During the conversion of muscle to meat, the inter-organ exchange of metabolites is interrupted and the biochemical processes defining meat quality depend on the metabolic state of the animal during slaughtering. The metabolic state of an organ or a tissue results from many endogenous and exogeneous factors like management system, feeding, stress, sex among many more. It has been shown that metabolite profiles could be used to differentiate between different species or non-related breeds by using blood or meat samples [19,22,33], emphasizing for the impact of genetics on the metabolic phenotype. However, a study using hot muscle and liver samples of close related genotypes in pigs had not yet been applied.

In this study, we performed a metabolite profiling approach with GC × GC qMS on blood and hot muscle and liver tissues to elucidate basic variations in metabolite profiles between individual pigs. We included two genotypes, each with gilts and boars to look at the impact from the gender and genetic background. The unbiased statistical analysis by PCA revealed that the diversity between individuals predominated the sexual and genetic predisposition as shown in the PCA plots in Figure 2. As exogenous factors like feed, management system and transport stress to the slaughter house were minimized it can be concluded that various endogenous factors like genotype, gender, individual stress feeling or health status were mutually affected causing the high variability of metabolite profiles. The classification by OPLS-DA revealed gender dependent differences, and further univariate statistical testing showed that among the multitude of analytes a substantial number was significantly different. Interestingly, more gender-dependent differences were obtained in the PIxGL background compared to the PIx(LWxGL) crossings. The obtained patterns of significantly different signals indicated that the abundance of molecules is not necessarily a characteristic of either genotype or sex but a combination of both. This became particularly clear for features that showed a difference only in one group compared to the other three—for example, the elevated blood fumarate levels in PIxGL boars (Figure 3). In the past, gender differences have mainly been discussed regarding boar taint, and differences in fat composition and fatty acid profiles, which affect consumers perception and technical processing to meat products [34,35,36,37]. Bovo et al., 2015 [38] applied a large study on mature female and castrated Italian Large White pigs. They targeted 186 metabolites and found differences in various metabolic pathways from which they suggested a metabolic shift in castrated males towards energy storage and lipid production. This indicated that feeding strategies could be adopted accordingly [38]. In this direction, considering the lower carcass weights and the lower meat areas in boars observed here, we assume that the feeding efficiency did not meet the required levels for optimal growth of the boars. This is also reflected in metabolic differences from which we suspect a shift in carbon and nitrogen metabolism (as discussed below).

In our study, the pigs were adolescent and sexual development was not yet completed, so that most probably the sexual divergences were not completely manifested in the metabolite profiles. The higher number of differentiating features between gilts and boars in the PIxGL background was observed for all three sample types. It can be suspected that the crossing of PI with a LW containing mother line in PIx(LWxGL) either generally leads to less pronounced metabolic variations between gilts and boars or that the reduced differences are related to developmental shifts, e.g., a later puberty stage and therefore even less sex-induced metabolic variance. It is well known that, during early development, the growth patterns of gilts and boars are very similar until puberty, which is about the slaughter age here [39,40]. During puberty, sex hormone induced physiological switches generally lead to increased fat deposition in sows and a higher muscle proportion in boars. It might be suspected that, at maturity, the metabolic phenotypes are more distinct.

The differences between both genotypes were less pronounced than the gender-dependent differences. Furthermore, the differences were either detected in gilts or boars. It appears that the LW containing mother line has only a small impact for genotype-dependent metabolic variations at this developmental stage. Other metabolomic studies, which used mature pigs, have shown that metabolite profiles between non-related breeds are more pronounced [22,23]. In both studies the authors could differentiate both genotypes by applying multivariate statistics, though their results also showed a considerable variability of individuals. Using univariate statistics, Carmelo et al., 2020 [23] detected only five metabolites that largely contributed to the discrimination between Duroc and Landrace pigs, and were significantly different. Bovo et al., 2016 [22] detected six significantly different metabolites between Duroc and Large white pigs. It can therefore be assumed that the genetic distance does not have to be a prerequisite for the strength of metabolic differentiation.

Taken together, our data indicate that genetics, gender and developmental stage cannot be considered in isolation for understanding physiological processes involved in breed metabolic characterization and differentiation. It would be interesting to elucidate to which extent the genetic distance impacts the metabolic separation in dependence of the developmental stage.

### 3.2. Carbon and Nitrogen Shifts in PIxGL Gilts and Boars

**Figure 6:**
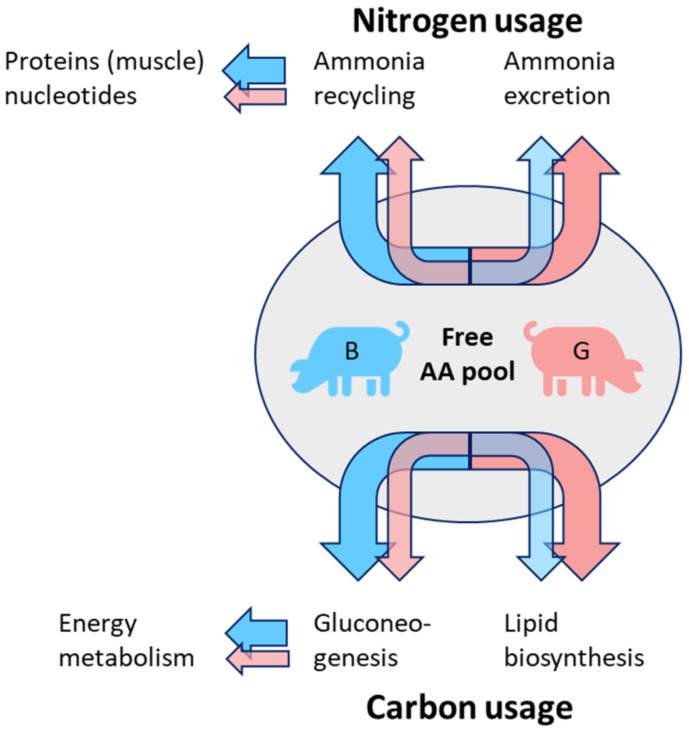
Proposed differences in amino acid metabolism between gilts and boars in the genotype PIxGL. According to the results from all three tissues, different pathways for C and N distribution are assumed. Whereas in boars (B) the ammonia is preferentially recycled and shuffled into protein biosynthesis and energy nucleotides, in gilts (G) the excretion level in the form of urea is higher. Released carbon skeletons are assumed to be preferentially shuffled into gluconeogenesis in boars to provide energy in gilts, more carbon might go into lipid biosynthesis.

Figure 6 summarizes our conclusions gained from metabolite profiles of all three sample types. Even though the pigs were adolescent at slaughtering, we already observed different tendencies for carbon and nitrogen use, especially in the PIxGL background. Tendentially, the amino acids that accumulated in gilts belonged to ketogenic as well as glucogenic amino acids, whereas, in boars, only the glucogenic amino acids glutamine, glutamate, and aspartate accumulated to a higher amount. Additionally, the glucogenic proline and its derivatives hydroxyproline and oxoproline were higher abundant in boars.

Glutamate and aspartate are key components in the interplay of TCA cycle, gluconeogenesis as well as amino acid redirection and degradation via urea cycle. Cytosolic aspartate is converted with citrulline to argininosuccinate, which is the branching point between urea cycle and TCA cycle, with the latter providing malate for the malate-aspartate shuttle and providing oxaloacetate for gluconeogenesis. Both mechanisms are highly active in liver. Aspartate is recovered from fumarate and glutamate via transamination catalysed by the aspartate transaminase (AST). Even though aspartate links the TCA cycle and urea cycle, and provides an amino group in urea synthesis, the urea levels in boars were not increased but tendentially lower than in gilts (Supplementary Table S2). The malate-aspartate shuttle (most active in liver, kidney, heart) is mostly important for the indirect transfer of cytosolic NADH into mitochondria to provide reduction equivalents needed during oxidative phosphorylation. In accordance with the higher aspartate levels in boars, malate was also accumulated in PIxGL boars (Supplementary Table S2). This might hint at a shunt in energy metabolism in PIxGL boars. Aspartate can reversibly be converted to glutamate catalysed by the Aspartate aminotransferase (AST), but no difference for glutamate or glutamine were observed in the liver.

Glutamate, which is more abundant in boar blood, is formed by deamination of glutamine, protein breakdown or transamination of alanine and α-ketoglutarate (Cahill cycle). From our results, we suspect that, in PIxGL boars, the recycling of ammonia by glutamine synthase (GS) is higher compared to gilts. The recycled ammonia could be used in protein anabolism such as muscle development. Vice versa, more ammonia is converted to urea and excreted in gilts. Glutamine, which is generally highly abundant in blood represents a multi-functional metabolite in many tissues because it is the main transport and storage form of ammonia, nitrogen donor for pyrimidine and purine synthesis, and it is important for protein anabolism, energy metabolism and defence (GSH precursor). Glutamine correlated very high to hydroxyproline, which is a major component of collagen. The additional association of hydroxyproline to glutamate and TCA components gave rise to the assumption that collagen synthesis was increased in boars. In this context, a high hydroxyproline pool was observed during muscle growth in relation with accompanied connective tissue turnover [41].

Regarding GSH homeostasis, levels of the GSH regulatory component 2-aminobutyric acid (2-AABA) in combination with oxoproline levels are discussed as potential biomarkers for GSH status because 2-AABA is a precursor of ophthalmate, which is an analog of GSH in which the L-cysteine is replaced by 2-AABA [42,43]. Therefore, it could be speculated that the high levels of 5-oxoproline and 2-AABA point towards reduced levels of GSH in boars compared to gilts, which could result from increased energy metabolism and thus a higher need for ROS scavenging. Furthermore, the higher levels of 2-hydoxybutyric acid in boars are additional hints for increased needs of GSH.

Concerning carbon metabolism, we speculate that a deamination of glucogenic amino acids in boars shuffles the carbon skeleton (pyruvate) into gluconeogenesis to provide glucose for the export to organs of consumption (e.g., energy metabolism in muscles). The lower levels of hexoses like fructose and glucose in muscle and higher levels of sugars in blood are probably a hint for higher sugar turnover rates in boars. An increased level of energy metabolism in PIxGL boars was indicated by the notable enhanced levels of TCA components in correlation with GA3P (Glycolysis), inosine and hypoxanthine (both breakdown products from ATP). The TCA cycle connects various metabolic pathways. Among them it is the intermediate section between glycolysis and oxidative phosphorylation (ATP production). The adenine nucleotide degradation products hypoxanthine and inosine significantly accumulated in boar blood, which substantiated our conclusion of increased energy metabolism in boars. Whether the hypoxanthine is used for adenine nucleotide restoration via the salvage pathway is not known. It has been described that increased levels of hypoxanthine and inosine can be linked to hypoxia and increased oxidative stress [44,45]. The correlation of ß-Alanine to0020hypoxanthine and citrate was a further indicator for higher ROS scavenging needs in boars. ß-Alanine is precursor of the dipeptide carnosine, which is discussed to exert antioxidant activity inhibiting lipid oxidation, and it may be a free radical scavenger and pH buffer [46].

In contrast to the proposed shift of the deaminated carbon skeleton into energy metabolism in boars, the ketogenic amino acids in gilts can be broken down to Acetyl-CoA, branching point into lipid biosynthesis. Lipogenesis is again closely related to energy metabolism. Therefore, correlations of compounds from lipid and energy metabolism in liver to meat quality factors might also reflect the importance of the liver for whole body homeostatic control. In general, different qualitative differences for lipid classes were observed in all three sample types. Among them, monoacylglycerols tended to higher levels in gilts. In relation to the proposed increased level of nitrogen excretion via the urea cycle in gilts the remaining carbon skeleton is supposed to feed into lipid biosynthesis. This is also supported by the higher fat amount in gilts. Interestingly, the fatty acids observed in this study showed a mixed pattern in intramuscular fat composition with saturated fatty acids higher accumulating in boars and non-saturated fatty acids higher accumulating in gilts.

Concluding from our results, we assume that, in boars (especially PIxGL), the re-use of nitrogen is enhanced compared to gilts. At the end of the fattening period, metabolism is increasingly shifted from nitrogen towards carbon (fat) metabolism. Our data indicate that the boars in this study are still showing considerable muscle growth. This is also reflected in Figure 1 (meat area and fat area). Furthermore, for the released carbon skeletons we suppose a stronger flow into gluconeogenesis in boars, whereas in gilts the carbons might be more channeled into lipogenesis. This would be in accordance with the assumption that the maturation in gilts is more progressed, and that gilt’s metabolism shows diminished muscle growth and more fat deposition. Further research should address development-dependent shifts of metabolites in pigs to draw more concrete conclusions about sexual dimorphism in energy metabolism and oxidative stress defence mechanisms. To prove our hypotheses, the elements of the Cory cycle, Cahill cycle and gluconeogenesis have to be analysed in detail. Additionally, the mechanisms of ROS scavenging and nucleotide metabolism should be elucidated. For this purpose, it is important to use pigs from different genetic backgrounds at different developmental stages.

### 3.3. Association of Metabolite Levels to Meat Quality Data

Pearson correlation and multiple regressions were calculated in order to estimate if the obtained metabolite markers (Table 1) are also associated with meat quality parameters, and if they might be interesting candidate compounds for the prediction of meat quality. In particular, metabolites from energy metabolism correlated to the various quality factors. This is accordance with Welzenbach et al., 2016 [21], who also observed also a high importance of energy metabolism in relation to drip loss. Muroya et al., 2014 [47] observed huge differences in energy-, amino acid—and nucleotide metabolism in slow and fast type muscles during aging, which indicates that the development of meat quality is also different depending on the muscle type and probably on consumption rates of energy metabolites. Considering the low variations in parameters describing meat quality of this sample set, no substantial contributions of candidate compounds were expected. Nevertheless, due to the close relationship between post-mortem glycolysis and the resulting meat quality, it is of interest if any relationship on this level can be detected.

The correlation of 1,5-Anhydroglucitol to the most important meat quality parameters (L*, pH1 and drip loss) predicting the resulting water binding capacity of meat, has not been described so far. This compound is described as a clinical marker of short-term glycemic control reacting on fast glucose fluctuations [48]. Thus, 1,5-Anhydroglucitol might represent an indirect marker for glucose consumption to fuel early post-mortem energy pathways. Similarly, liver acetyl glucosamine levels, which also correlated to water holding can be suspected to mirror the energetic status of the liver as these molecules play regulative roles in lipogenesis and gluconeogenesis/glycogenesis [49].

An early prediction of meat quality, for example by using sticking blood, would be very useful for slaughter house operators to optimize the post-mortem technological treatments like chilling on a more individual level. The observed moderate regression coefficients for pH1, drip loss, b* and fat area indicate that it might be worth using metabolomic data for a large-scale investigation, which would help to better understand relations between metabolic pathways, drivers and meat quality. So far, sticking blood has been used to detect differences according to the genetic background, to gender or feeding efficiency. Carmelo et al., 2020 [23] who used Duroc and Landrace pigs, confirmed the study from Bovo et al., 2016 [22] on the applicability of metabolomics to discriminate between (genetically distant) breeds. Bovo et al., 2015 [38] also showed differences between sows and castrates within one genotype. In addition, Carmelo et al., 2020 [23] linked metabolite profiles to feeding efficiencies in pigs and observed genotype-dependent responses to testing time and feeding type. Thus, concluding from our observations and results gained from other research, a prediction of final meat quality using sticking blood or muscle biopsy will hardly be achieved. A multifactorial network of influence factors, like genetic distance, gender, age, feeding and husbandry formulate individual in vivo metabolite profiles. Thus, comprehensive meta information would have to be dissected for each individuum in a slaughter line. Besides the pre-slaughter conditions, the processes during muscle-meat transition are also complex and manifold, and metabolite profiles are only one part of the story defining meat quality. Attempts made so far applying metabolomics to meat quality mainly focused on the use of meat (24 h after slaughter) in relation to aging [50,51,52,53] or development of sensory attributes [33,54]. Therefore, the usage of biomarkers might become relevant for particular questions of meat handling, for example the extent of ripening. Following the basic applications so far, future studies should elucidate the discriminative power for samples from different production systems or for different breeds and genders to validate the stated hypotheses.

## 4. Materials and Methods

### 4.1. Chemicals

All chemicals used were commercially obtained from Merck (Darmstadt, Germany) or Altmann Analytics (Munich, Germany), and are listed in Supplementary Table S3.

### 4.2. Sample Collection

The animals were reared under the same conditions at the Bavarian performance testing institute Schwarzenau, Germany. No particular treatments were conducted so that no ethical statements were needed. The pigs were reared for about 100 days in the test station until they reached their testing weight, a hot carcass weight of 95 kg. The pigs were fed with a two-phase feeding. In the first six weeks they were fed with diet 1. From week seven onwards diet 2 was fed. The compositions of the both diets are provided in Supplementary Table S4 [55,56]. The slaughterhouse was on-site, so that transport stress was minimal. The samples were randomly collected at an on-site slaughtering plant, and at three slaughtering days with an interval of one week. The sample set consisted of 40 pigs of two common German crossbreds, 18 PIx(LWxGL) (11 gilts and 7 entire males) and 22 PIxGL (10 gilts and 12 entire males).

The pigs were electrically stunned with a semi- automatic stunning device (Haas, Neuler, Germany) and the blood samples were collected during exsanguination. Muscle and liver samples were collected at the cutting line, approximately 30 min after exsanguination. Muscle samples were taken from left *M. longissimus dorsi* between the 12 and 13 ribs. Liver samples were taken from the left medial lobule. All samples were immediately frozen in liquid nitrogen and stored until further use at −80 °C.

### 4.3. Meat Quality Parameters

Meat quality parameters color and drip loss were analysed using a 3 cm thick cutting from the right *M. longissimus dorsi* at the 12th/13th rib, taken 24 h after slaughter. Color parameters L*, a*, b* were measured after 15 min bloom time with a Minolta Chroma Meter CR-310 (Konica Minolta, Munich, Germany). Drip loss was determined according to the EZ-DripLoss method from Rasmussen and Andersson (1996) [57]. Therefore, duplicate samples were punched out from each cutting with a Ø 2.5 cm circular knife and stored at 4 °C for 24 h in EZ drip loss containers.

PH1 measurement was performed 45 min post-mortem. The measurement was done in the *M. longissimus dorsi* between the 13th and 14th thoracic vertebrae in a depth of 4 cm with a pH probe. Ultimate pH was measured after 24 h in *M. longissimus dorsi* and *M. semimembranosus*.

The meat area is the surface of the *M. longissimus dorsi* at the cut part of the chop in cm^2^. The fat area is the fat surface above the cut part of chop in cm^2^. To measure the meat and fat area, the chops were cut on the hanging carcass at the level of the 13th and 14th thoracic vertebrae. This section was photographed and the measurement was carried out on the image (SCAN-STAR K, R.Matthäus, Eckelsheim, Germany).

For significance calculations we applied Tukey’s HSD test implemented in the JMP software (13.1.0, SAS Institute Inc., Cary, NC, USA). For association of selected analytes to meat quality parameters we calculated the Pearson correlation coefficient, and additionally applied a multiple regression to check for predictability. Both coefficients were also calculated in JMP software.

### 4.4. Sample Preparation and GC × GC qMS Measurement

Sample preparation and measurement were performed according to Wagner et al., 2020 [58]. In brief, all tissue samples were lyophilized and homogenized to powder with a bead mill before sample extraction. For GC × GC qMS analysis we included quality check samples (QC) and blank samples in addition to our biological study samples. The QC samples were prepared for each tissue type (whole blood, muscle and liver) and consisted of an aliquot from each sample, respectively. All samples were extracted in the same manner. The blank samples didn’t contain any matrix. Firstly, 20 mg of the dried, homogeneous powder were extracted with 600 µL ice-cold 80% methanol containing an internal standard mix (Supplementary Table S3) using a bead mill homogenizer (Minilys, Bertin Technologies SAS, France) and subsequently an ultrasonic bath for 2 min. After centrifugation the pellet was re-extracted with 600 µL ice-cold methanol:chloroform (2:1 *v*/*v*) according to the first extraction step. The combined supernatants were transferred into 2 mL glass vials containing a 200 µL glass insert, dried in a vacuum centrifuge (Christ Speedvac RVC 2-18 CD plus, Germany) and finally stored under protective argon atmosphere at −80 °C until analysis.

Before measurement samples were derivatized, methoximation was carried out using a 20 mg/mL solution of MAH in pyridine at 50 °C for 1 h. In a second step, 70 µL of MSTFA + 1% TMCS were added and samples were shaken at 70 °C for 1h. The measurements were carried out on a Shimadzu GCMS QP2010 instrument (Shimadzu, Duisburg, Germany). Instrumentation and parameter details are provided in Supplementary Table S3. Full scan data were acquired in a mass range of 60–550 *m*/*z*.

### 4.5. Metabolomic Data Analysis

Peak integration of the acquired raw data was performed with GCMS Postrun Analysis Module within the instrument software GCMSsolution (Version 4.45, Shimadzu, Duisburg, Germany). Subsequently, peak quality filtering, peak alignment, signal intensity drift correction and quality assessment were performed [29,30]. For visualization of chromatograms and annotation of compounds we used the NIST 14 library database implemented in GC Image Software (Version 2.7, GC Image, Lincoln, NE, USA). A series of n-alkanes (C7-C30) was used as a retention time standard.

We applied two different approaches of multivariate data analyses, Principle Component Analysis (PCA) and Orthogonal Projections to Latent Structures Discriminant Analysis (OPLS-DA). Both are commonly used methods for multivariate analysis (MVA) in high dimensional data space, such as metabolomics. PCA is an unbiased unsupervised dimension reduction method, which does not include any class information, while OPLS is a supervised MVA dimension reduction method, which incorporates the provided class information with the ultimate goal to differentiate the groups from different classes (discriminant analysis) on the basis of the provided data variables matrix. Conceptually, the data matrix is decomposed into scores and loadings vectors. For the supervised method, variable importance projections (VIP) are used to extract meaningful variables (in our case analytes), being important (VIP > 1) for an appropriate class segregation [59]. MVA was performed using the SIMCA-P+ software (version 13, Umetrics, Umeå, Sweden). All variables were centered and unit variance scaled. In order to visualize general differences among the sample groups (two genotypes, each with gilts and boars) and test for outliers we applied in a first step PCA. Hotelling’s T2 Range was used to find samples lying outside the critical limits of the model plane (0.05 level). These were treated as outliers and removed from further analysis. We removed 2 samples from muscle and one sample from blood analyses. In a second step we performed OPLS-DA to analyze for differences between genders and genotypes.

For univariate statistical calculations we used the JMP software (13.1.0, SAS Institute Inc., Cary, NC, USA). In a first step we used the Anderson Darling Test to check for normal distributions of analytes. Then we applied Tukey’s HSD Test (for normal distributed variables) and Steel-Dwass Test (for non-parametric variables) to elucidate significant differences (*p* < 0.05) of analytes between genders and genotypes. Both include a correction for family-wise error rates by multiple testing. In addition, we also show results (*p* < 0.05) from simple Student’s *t*-test (for normal distributed variables) and Wilcoxon test (for non-parametric data) to also take analytes into account that show tendencies for a differentiation between sample groups (but are probably not strong enough due to the size of the sample set).

The heatmap illustration in Figure 5 was created in MetaboAnalyst (https://www.metaboanalyst.ca/home.xhtml, accessed on 22 January 2021) [60]. For integration of the identified compounds into metabolic pathways we used information from KEGG (https://www.genome.jp/kegg/pathway.html, accessed on 22 January 2021) and HMDB (https://hmdb.ca/, accessed on 22 January 2021) databases.

## 5. Conclusions

As key outcomes of this study we concluded that the sexual metabolic dimorphism depends on the genetic background and vice versa that metabolic differences between genotypes depend on gender. We observed substantial patterns related to gender differences, especially in the PIxGL background, from which we deduced hypotheses for differences in carbon and nitrogen shunt. We concluded that released ammonia from the free amino acid pool in gilts is excreted to higher amounts via urea, whereas in boars a higher rate of N-recycling is presumed. Released carbon skeletons from deaminated amino acids might be shuffled to a higher extent into gluconeogenesis in boars compared to gilts, while in gilts we assume a higher use of released carbon skeletons for lipogenesis.

Blood as the transport medium between the different organs partly reflected the metabolic levels of muscle tissue samples. From a practical point of view, a system which could predict the resulting meat quality already from blood samples at exsanguination, would, for example, allow for adjusting carcass handling practices. However, with the current study, we demonstrate that each animal has to be considered as an individual reflected by the variability of metabolite patterns in all three tissues. Furthermore, correlations to selected carcass and meat quality parameters did not result in a strong predictability. We point out that the use of sticking blood or hot muscle samples has limited applicability for meat quality prediction. Besides internal factors (gender, age, breed) and pre-slaughter conditions, the processes during muscle-meat transition are also complex and manifold. It appeared that holistic considerations are needed. In this direction further research should at least take time, genetic distance and genders into account when relating metabolite profiles to performance data.

## Figures and Tables

**Table 1:** Numbers of candidate molecules that potentially play a role in sexual metabolic dimorphism or genotype differentiation. VIP—Variable Importance Projection.

		Blood	Muscle	Liver
	Signals used in data analysis	375	323	476
Differences between gilts and boars	VIP > 1	117	89	120
	Significant by Student’s T-Test/Wilcoxon	61	41	75
	Significant by Tukey’s HSD/Steel Dwass Test	26	9	28
Differences between both genotypes	Significant by Student’s T-Test/Wilcoxon	15	15	20
	Significant by Tukey’s HSD/Steel Dwass Test	3	2	5

**Table 2:** Significant differences between gilts and boars regarding dependence on the genotype. According to the outcomes from Anderson-Darling’s Test for normal distribution the results from Tukey HSD Test or Steel-Dwass Test have to be considered. A data table with candidate features from T-Test/Wilcoxon Test and OPLS-DA (VIP scores) analysis is provided in Supplementary Table S2; n.a.—not applied due to limit of detection; FC—fold change.

Metabolite Class	Tissue	Analyt ID	Annotation	OPLS-DA	Test for Normality	Genotype	Parametric	Non-Parametric	log2 FC (Boars/Gilts)
				VIP	Anderson-Darling		Tukey	Steel-Dwass	
Amino acids and derivatives	Blood	A0274	Beta-alanine	2.66655	<0.0001	PIxGL	0.0135	0.0199	0.95
		A0363	Oxoproline	2.14204	0.785	PIxGL	0.0378		0.30
		A0381	Hydroxyproline	1.32486	0.025	PIxGL	0.0288	0.0389	0.48
		A0386	Unknown amine	1.87376	<0.0001	PIxGL		0.0389	1.25
		A0462	Glutamic acid	1.72612	0.316	PIxGL	0.0361	0.0389	0.35
		A0528	Glutamine	1.63493	<0.0001	PIxGL	0.0341	0.0251	0.66
		A0581	Putative histamine	1.69071	0.528	PIxGL	0.0443		0.49
	Muscle	A0273	Hydroxyproline	2.01281	0.132	PIxGL	0.0047	0.0074	0.42
		A0449	Glutamine	2.39137	0.142	PIxGL	0.0135	0.0147	0.46
	Liver	A0173	Putative allo-Isoleucine		0.325	PIx(LWxGL)	0.0213		−0.65
		A0328	Aspartic acid	3.32175	0.038	PIx(LWxGL)	0.0014	0.0155	0.64
		A0328	Aspartic acid	3.32175	0.038	PIxGL	0.0007	0.0085	0.65
		A0333	Unknown amine	2.1025	0.814	PIxGL	0.0416		0.26
		A0502	Phosphorylethanolamine	1.43293	0.673	PIxGL	0.0073	0.0067	0.70
		A0680	Acetyl glucosamine 1	1.94297	0.001	PIxGL	0.0189	0.0468	−1.20
		A0686	Acetyl glucosamine 2	1.96005	<0.0001	PIxGL	0.0152	0.0126	−1.89
Lipids	Blood	A0483	Putative fatty acid	1.79711	0.473	PIxGL	0.0386		0.66
		A0624	Fatty acid (putative Pentadecanoic acid)	2.18734	0.001	PIxGL	0.015	0.0033	0.44
	Liver	A0808	Eicosatrienoic acid	2.08445	0.001	PIx(LWxGL)		0.0487	1.94
		A0816	Fatty acid (putative Butyl-9,12-octadecadienoate)	2.2954	0.004	PIx(LWxGL)		0.0478	0.27
		A0847	Eicosapentaenoic acid	2.2939	0.066	PIxGL	0.0013	0.0107	−0.51
Organic acids	Blood	A0430	2-Oxoglutaric acid	1.75833	<0.0001	PIxGL	0.0097	0.0199	1.06
		A0453	Putative pimelic acid		0.06	PIxGL	0.0248	0.0199	0.20
		A0565	Citric acid	2.46605	<0.0001	PIxGL	0.0009	0.0251	1.03
	Muscle	A0481	Citric acid	2.05545	0.257	PIxGL	0.0205	0.0277	1.10
	Liver	A0205	Fumaric acid	1.52228	0.419	PIxGL	0.0296	0.0107	0.98
Carbo-hydrates	Blood	A0497	Pentose (unknown isomer)	1.54153	<0.0001	PIxGL	0.0162	0.0478	1.23
		A0558	Glyceraldehyde 3-phosphate	1.77768	0.636	PIxGL	0.0059	0.0251	0.76
		A0618_H2	Hexose alcohol (unknown isomer)	1.51685	0.47	PIxGL	0.0018	0.0313	0.64
		A0656	Myo Inositol	1.81065	0.719	PIxGL	0.0385		0.28
	Muscle	A0489	1,5-Anhydroglucitol	2.31495	0.516	PIxGL	0.0412	0.0339	−0.82
	Liver	A0624	Inositol (unknown isomer)	1.51716	0.18	PIx(LWxGL)	0.0315		−0.79
		A0659	Inositol (unknown isomer)	1.95406	0.106	PIxGL	0.0316		0.32
		A0778	Glyceryl-glycoside	2.24976	0.129	PIxGL	0.0002	0.0079	1.91
Nucleotide metabolism	Blood	A0550	Hypoxanthine	2.29192	<0.0001	PIxGL	0.0027	0.0033	1.09
		A0731	Inosine	1.50638	<0.0001	PIxGL		0.031	n.a.
Unknowns	Blood	A0237	Unknown	1.44216	0.005	PIx(LWxGL)		0.0478	0.17
		A0521	Unknown	2.15177	0.141	PIx(LWxGL)	0.0425		−0.71
		A0135	Unknown	2.31982	0.035	PIxGL	0.0326	0.048	0.95
		A0336	Unknown	1.35796	0.171	PIxGL	0.0432	0.0124	0.36
		A0432	Unknown	1.25233	<0.0001	PIxGL	0.0449	0.0217	1.14
		A0467	Unknown	1.19814	<0.0001	PIxGL		0.038	2.20
		A0590	Unknown	1.92151	<0.0001	PIxGL		0.048	1.46
		A0603	Unknown	1.6805	<0.0001	PIxGL		0.048	1.76
	Muscle	A0431	Unknown	1.37717	0.423	PIx(LWxGL)	0.0138		0.34
		A0013	Unknown	1.28436	0.107	PIxGL	0.0496		−0.16
		A0428	Unknown	2.46274	0.310	PIxGL	0.0107	0.0498	0.55
		A0555	Unknown	1.8396	0.500	PIxGL	0.0451		1.18
		A0714	Unknown	2.56177	0.040	PIxGL	0.0044	0.0116	1.57
	Liver	A0076	Unknown	2.24581	0.643	PIx(LWxGL)	0.0046	0.0478	−0.65
		A0266	Unknown		0.456	PIx(LWxGL)	0.0383		−0.66
		A0533	Unknown	2.06327	0.654	PIx(LWxGL)	0.0287	0.0277	−0.62
		A0569	Unknown	1.4654	0.814	PIx(LWxGL)	0.0275		−0.65
		A0608	Unknown	1.50611	0.004	PIx(LWxGL)	0.0451	0.0478	−0.90
		A0615	Unknown	2.11088	0.607	PIx(LWxGL)	0.0129		−1.12
		A0862	Unknown	1.19302	0.058	PIx(LWxGL)	0.0186		−0.90
		A0356	Unknown		0.144	PIxGL	0.0443		0.55
		A0364	Unknown	1.61973	0.049	PIxGL	0.0193	0.0168	1.12
		A0379	Unknown	2.50225	0.776	PIxGL	0.0283	0.0209	−0.48
		A0447	Unknown	1.02693	<0.0001	PIxGL		0.0117	2.31
		A0528	Unknown	1.21156	0.24	PIxGL	0.0123	0.0386	1.20
		A0536	Unknown		0.879	PIxGL	0.0359		0.49
		A0610	Unknown	2.35537	0.005	PIxGL		0.0423	−1.71
		A0795	Unknown	1.89984	0.413	PIxGL	0.0155	0.0316	0.86

**Table 3:** Correlations of energy balance related metabolites and compounds connected to tissue protection. The upper right corner contains *p*-values for the correlation probability and the lower left corner the correlation coefficients (r). In addition, the correlation is visualized with a color gradient from r = −1 (dark blue), gray intermediate to r = 1 (red).

	Beta-Alanine	Oxoproline	Hydroxy-proline	GA-3P	Citrate	2-Oxoglutarate	Glutamate	Glutamine	Hypoxanthine	Inosine	2-Hydroxybutyric Acid	2-Ainobutyric Acid	Cysteine
Beta-Alanine		0.0040	0.0078	0.0051	0.0003	0.0334	0.2128	0.0341	0.0005	0.0093	0.9290	0.5147	0.2276
Oxoproline	0.4681		<0.0001	0.0025	<0.0001	0.0208	0.0017	<0.0001	0.0515	0.1316	0.9447	0.6478	0.0625
Hydroxyproline	0.4365	0.7306		0.0060	0.0019	0.0370	0.0122	<0.0001	0.3070	0.6759	0.7480	0.4436	0.1208
GA3P	0.4566	0.4889	0.4488		<0.0001	0.0007	0.1427	0.0005	0.0003	0.0420	0.0182	0.0419	0.0085
Citrate	0.5669	0.6664	0.4995	0.6718		<0.0001	0.0001	<0.0001	<0.0001	0.0019	0.1199	0.3042	<0.0001
2-Oxoglutarate	0.3555	0.3838	0.3489	0.5403	0.7480		0.0011	0.0198	<0.0001	0.0064	0.0024	0.0197	<0.0001
Glutamate	0.2128	0.5049	0.4133	0.2492	0.5992	0.5211		0.0004	0.1037	0.1478	0.5975	0.6655	0.0013
Glutamine	0.3541	0.8134	0.6822	0.5476	0.6974	0.3866	0.5573		0.0750	0.0965	0.8977	0.6672	0.0823
Hypoxanthine	0.5484	0.3271	0.1751	0.5727	0.6717	0.6893	0.2757	0.3005		<0.0001	0.0135	0.1196	0.0010
Inosine	0.4274	0.2561	0.0721	0.3408	0.5003	0.4460	0.2462	0.2813	0.8147		0.4198	0.9585	0.0643
2-Hydroxybutyric acid	0.0154	−0.0120	−0.0555	0.3916	0.2639	0.4905	0.0910	−0.0222	0.4079	0.1387		<0.0001	<0.0001
2-Aminobutyric acid	−0.1122	−0.0788	−0.1318	0.3408	0.1761	0.3870	0.0746	−0.0742	0.2641	0.0090	0.8957		<0.0001
Cysteine	0.2062	0.3136	0.2633	0.4322	0.6387	0.8274	0.5154	0.2935	0.5270	0.3116	0.7515	0.6212	

**Table 4:** Pearson correlation coefficients (r) of quality parameters to compounds that showed significant differences between boars and gilts, and multiple regression coefficients with predictors having an r > 0.4 (at least moderate correlation). For the explained variance [R^2^] the adjusted value is presented.

Tissue	Quality Parameter	Compound	Correlation Coefficient [r]	Significance Probability [p]	Explained Variance [adj. R^2^]	Significance of the Model [p]
Blood	% Drip loss	Hexose alcohol (A0618_H2)	−0.329	0.047		
	b*	Putative fatty acid (A0483)	−0.466	0.004	0.28	0.0085
	b*	Hypoxanthine	−0.439	0.007		
	b*	Glyceraldehyde 3-phosphate	−0.407	0.013		
	b*	Beta-alanine	−0.406	0.013		
	b*	Unknown (A0237)	−0.400	0.014		
	b*	Unknown (A0135)	−0.362	0.028		
	b*	Citric acid	−0.358	0.030		
	b*	Inosine	−0.350	0.034		
	L*	Citric acid	−0.350	0.034		
	pH 1	Putative histamine	0.385	0.019		
	pH 1	Hexose alcohol (A0618_H2)	0.349	0.034		
	Fat area	Unknown (A0135)	−0.520	0.001	0.27	0.0094
	Fat area	Glutamine	−0.430	0.008		
	Fat area	Citric acid	−0.417	0.010		
	Fat area	Putative histamine	−0.402	0.014		
	Fat area	Beta-alanine	−0.400	0.014		
	Meat area	Unknown (A0135)	−0.329	0.047		
Muscle	% Drip loss	Citric acid	−0.463	0.004	0.32	0.0005
	% Drip loss	1,5-Anhydroglucitol	0.569	0.000		
	b*	Citric acid	−0.421	0.010	0.15	0.0095
	b*	Unknown (A0428)	−0.362	0.028		
	L*	Citric acid	−0.337	0.042		
	L*	1,5-Anhydroglucitol	0.508	0.001	0.23	0.0013
	pH 1	1,5-Anhydroglucitol	−0.501	0.002	0.27	0.0018
	pH 1	Citric acid	0.471	0.003		
	pH 1	Glutamine	0.346	0.036		
Liver	% Drip loss	Fumaric acid	−0.366	0.024		
	% Drip loss	Unknown (A0447)	−0.341	0.036		
	% Drip loss	Acetyl glucosamine 2	0.325	0.046		
	% Drip loss	Acetyl glucosamine 1	0.325	0.047		
	a*	Unknown (A0076)	0.403	0.012	0.28	0.0112
	a*	Inositol (A0624)	0.401	0.013		
	a*	Unknown (A0608)	0.387	0.016		
	a*	Aspartic acid	−0.361	0.026		
	b*	Aspartic acid	−0.543	0.000	0.31	0.0006
	b*	Unkown (A0795)	−0.436	0.006		
	b*	Unknown (A0533)	0.378	0.019		
	b*	Fatty acid (A0816)	−0.373	0.021		
	b*	Unknown (A0615)	0.342	0.036		
	b*	Eicosatrienoic acid	−0.330	0.043		
	b*	Fumaric acid	−0.325	0.047		
	b*	Inositol (A0659)	−0.323	0.048		
	b*	Unknown (A0610)	0.321	0.049		
	b*	Eicosapentaenoic acid	0.320	0.050		
	L*	Eicosapentaenoic acid	0.521	0.001	0.25	0.0078
	L*	Acetyl glucosamine 2	0.424	0.008		
	L*	Acetyl glucosamine 1	0.408	0.011		
	L*	Fumaric acid	−0.403	0.012		
	L*	Unknown (A0528)	−0.324	0.048		
	pH 1	Unknown (A0528)	0.376	0.020		
	pH 1	Glyceryl-glycoside	0.344	0.035		
	pH 1	Acetyl glucosamine 2	−0.329	0.044		
	pH 1	Acetyl glucosamine 1	−0.323	0.048		
	Fat area	Unknown (A0533)	0.488	0.002	0.34	0.0038
	Fat area	Unknown (A0379)	0.431	0.007		
	Fat area	Acetyl glucosamine 2	0.425	0.008		
	Fat area	Acetyl glucosamine 1	0.416	0.009		
	Fat area	Urea	0.405	0.012		
	Fat area	Unknown (A0615)	0.403	0.012		
	Fat area	Eicosapentaenoic acid	0.393	0.015		
	Fat area	Fatty acid (A0816)	−0.368	0.023		
	Fat area	Fumaric acid	−0.358	0.027		
	Fat area	Eicosatrienoic acid	−0.335	0.040		
	Fat area	Unknown (A0569)	0.322	0.049		
	Meat area	Aspartic acid	−0.389	0.016		
	Meat area	Acetyl glucosamine 2	0.377	0.020		
	Meat area	Acetyl glucosamine 1	0.369	0.023		
	Meat area	Unknown (A0615)	0.345	0.034		

## Data Availability

Data is contained within the article or supplementary material.

## Supplementary Materials

The following are available online at https://www.mdpi.com/article/10.3390/metabo11050261/s1, Supplementary Table S1: Carcass and meat quality data; Supplementary Table S2: Overview of molecular features as obtained by univariate statistical testing and OPLS-DA analysis; Supplementary Table S3: Chemicals and measurement parameters for GC × GC qMS analysis. Supplementary Table S4: Composition of the feeding diet. Supplementary Table S5: GC × GX qMS preprocessed data used for statistical analysis.
