# Supplementary material for: Sexual Dimorphism of Metabolite Profiles in Pigs Depends on the Genetic Background
Source: Metabolites. 2021 Apr 22;11(5):261. doi: 10.3390/metabo11050261 (PMC8146355; doi:10.3390/metabo11050261)
Supplement: Supplementary file 1 [file metabolites-11-00261-s001.zip › Supplemental Table S3 - Chemicals-Parameters.pdf]

GC x GC qMS Analysis

Chemicals

Chemical	Purity	Supplier	Used for
Methanol	Hypergrade LC/MS	Merck	Extraction
Chloroform	p. A.	Merck	Extraction
Heptane	HPLC	VWR	Washing solution GC
Aceton	picograde	Promochem	Washing solution GC
Hexane	HPLC	Roth	Dilution Alkane
Pyridine	99,8%	Sigma-Aldrich	Derivatisation
N-Methyl-*N*-(trimethylsilyl)trifluoroacetamide (MSTFA) with 1 % trimethylchlorosilane (TMCS)		Thermo Scientific	Derivatisation
O-Methoxylamine hydrochloride (MAH)	GC	Sigma-Aldrich	Derivatisation
Alkane	Certified reference standard	Supelco	Retention Standard
2-Chlorphenylessigsäure	At least 95% purity	Alfa Aesar	Internal Standard
5-Brom-2,4-dihydroxybenzoesäure		Acros Organics	Internal Standard
p-Chlorphenylalanin		Acros Organics	Internal Standard
Phenyl beta-D-glucopyranoside hydrate		Sigma-Aldrich	Internal Standard
Pinitol		Alfa Aesar	Internal Standard
Sucralose		Alfa Aesar	Internal Standard
Adonitol		Sigma	Internal Standard
Deoxy-Ribose		Aldrich	Internal Standard
Bromooctadecane		Aldrich	Internal Standard
13C-Glucose		Aldrich	Internal Standard
Dibromophenol		Supelco	Internal Standard

Internal Standard Mixture

Internal Sandard	Concentration in first extraction solution (80% Methanol)
2-Chlorophenyl acetic acid	20 µM
5-Brom-2,4-dihydroxybenzoic acid	35 µM
p-Chloro-phenylalanine	50 µM
Phenyl-β-D-glucopyranoside	10 µM
Pinitol	10 µM
Sucralose	10 µM
Adonitol	10 µM
Deoxy-Ribose	20 µM
Bromooctadecane	50 µM
13C-Glucose	20 µM
Dibromophenol	20 µM

Instrumentation and Software

Instruments and software	Name	Manufacturer
Gas chromatograph	GC-2010	Shimadzu Corp, Kyoto, Japan
Mass spectrometer	QP2010 Ultra	Shimadzu Corp, Kyoto, Japan
Auto sampler	AOC-20s	Shimadzu Corp, Kyoto, Japan
PTV Injector	OPTIC-4	GL Sciences, Eindhoven, The Netherlands
Modulator	Zoex ZX1	ZOEX Corp., Houston, USA
GCMS instrument software	GCMS Solution 4.45	Shimadzu Corp, Kyoto, Japan
PTV software	Evolution Workstation 4.1	GL Sciences, Eindhoven, The Netherlands
GC×GC visualization software	GC Image 2.7	GC Image, LLC, Lincoln, Nebraska

Measurement parameters

Parameter	Setting / value
	GC parameters
Carrier gas	Helium
Liner	Deactivated borosilicate glass liner (ID 3.4 mm) with quartz wool (CS-Chromatographie)
^{1}D column	Rxi-5Sil MS, L = 30 m plus 10 m Integra Guard column, ID = 0.25 mm; film thickness = 0.25 µm (Restek)
^{2}D column	BPX50, L = 2.6 m, including a “separation segment” of L = 1.1 m, ID = 0.15 mm, film thickness = 0.15 µm (SGE)
Column connector	SilTite MicroUnion (SGE)
GC temperature ramp (blood)	80.0°C →2.5°C/min→150°C→3.0°C/min→240°C→17°C/min→320°C (hold 7min); total run time 69.7 min
GC temperature ramp (muscle)	90.0°C →3.0°C/min→100°C→2.2°C/min→200°C→3.0°C/min→260°C → 5.0°C/min→ 320°C (hold 5min); total run time 85.8 min
GC temperature ramp (liver)	80.0°C →3.0°C/min→180°C→2.5°C/min→300°C→6.0°C/min→320°C (hold 5min); total run time 89.7 min
GC mode	Constant velocity (35)
Injection mode	Split
Injection volume	1.0 µL
Split ratio	blood: 1:3; muscle: 1:15; liver: 1:10
PTV temperature ramp	40°C→10°C/s→280 °C, hold until end of run
Interface temperature	280 °C
	Modulation parameters
Modulator type	Cryogenic, air-based, loop-type
Modulation period (P_M)	Blood: 2.9 s; muscle: 4.5s; liver: 4.5s
Cold jet flow	8 l/min
Hot jet temperature	Programmed stepwise, at least 50 °C above oven temperature until 350°C
Hot jet duration	375 ms
	MS parameters
Ion source temperature	200 °C
Ionization mode	EI (70 eV)
MS Mode	Scan
Scan speed	20.000 amu/s
Scan range	m/z 60-550
Event time	30 ms
Data acquisition frequency	33 s^{-1}
